# Communicating cardiovascular disease risk: an interview study of General Practitioners’ use of absolute risk within tailored communication strategies

**DOI:** 10.1186/1471-2296-15-106

**Published:** 2014-05-29

**Authors:** Carissa Bonner, Jesse Jansen, Shannon McKinn, Les Irwig, Jenny Doust, Paul Glasziou, Kirsten McCaffery

**Affiliations:** 1 Screening and Test Evaluation Program (STEP), Sydney School of Public Health, Edward Ford Building A27, The University of Sydney, Sydney, NSW 2006, Australia; 2 Centre for Medical Psychology and Evidence-based Decision-making (CeMPED), Edward Ford Building A27, The University of Sydney, Sydney, NSW 2006, Australia; 3 Faculty of Health Sciences and Medicine, Bond University, Robina, QLD 4226, Australia

**Keywords:** Cardiovascular disease risk, Prevention, General practice, Primary care, Doctor-patient communication, Risk communication, Risk perception

## Abstract

**Background:**

Cardiovascular disease (CVD) prevention guidelines encourage assessment of absolute CVD risk - the probability of a CVD event within a fixed time period, based on the most predictive risk factors. However, few General Practitioners (GPs) use absolute CVD risk consistently, and communication difficulties have been identified as a barrier to changing practice. This study aimed to explore GPs’ descriptions of their CVD risk communication strategies, including the role of absolute risk.

**Methods:**

Semi-structured interviews were conducted with a purposive sample of 25 GPs in New South Wales, Australia. Transcribed audio-recordings were thematically coded, using the Framework Analysis method to ensure rigour.

**Results:**

GPs used absolute CVD risk within three different communication strategies: ‘positive’, ‘scare tactic’, and ‘indirect’. A ‘positive’ strategy, which aimed to reassure and motivate, was used for patients with low risk, determination to change lifestyle, and some concern about CVD risk. Absolute risk was used to show how they could reduce risk. A ‘scare tactic’ strategy was used for patients with high risk, lack of motivation, and a dismissive attitude. Absolute risk was used to ‘scare’ them into taking action. An ‘indirect’ strategy, where CVD risk was not the main focus, was used for patients with low risk but some lifestyle risk factors, high anxiety, high resistance to change, or difficulty understanding probabilities. Non-quantitative absolute risk formats were found to be helpful in these situations.

**Conclusions:**

This study demonstrated how GPs use three different communication strategies to address the issue of CVD risk, depending on their perception of patient risk, motivation and anxiety. Absolute risk played a different role within each strategy. Providing GPs with alternative ways of explaining absolute risk, in order to achieve different communication aims, may improve their use of absolute CVD risk assessment in practice.

## Background

Clinical practice guidelines for cardiovascular disease (CVD) prevention are based on absolute risk assessment to guide the use of preventive medication, rather than treating blood pressure and cholesterol as individual risk factors [1-3]. Various absolute risk models exist, but they are all based on the idea of using the most predictive risk factors, including non-modifiable risk factors such as age and gender, to assess the overall risk of a cardiovascular event over a certain period of time [3]. This represents a major shift in clinical practice, from assessing and treating blood pressure and cholesterol as independent risk factors, to managing overall CVD risk. Australian absolute risk guidelines are based on the widely used Framingham risk equation, where age, gender, smoking, diabetes, systolic blood pressure and cholesterol ratio are used to estimate the percentage risk of a cardiovascular event over the next 5 years [1,2,4]. Risk levels are defined as ranges of percentage risk, with different management recommendations for low (<10%), moderate (10-15%) and high (>15%) risk categories.

Reviews suggest the absolute risk approach may improve clinical management, patients’ risk perception and patients’ preventive intentions [5,6]. However, many General Practitioners (GPs) do not use absolute risk consistently for CVD risk assessment and management [7], and communication difficulties have been identified as one of the barriers to changing clinical practice [8,9]. GPs have also reported that they use absolute risk assessment tools if they think it will be useful for patient education [10]. The function of risk communication may therefore act as either a barrier to, or a facilitator of, the use of absolute risk guidelines.

A systematic review of CVD risk formats suggests that percentage and frequency formats are more effective than qualitative risk category descriptions or usual care for enhancing patient understanding of their risk and motivation to take preventive action [11]. This aligns with the shared decision making approach, which generally includes informing patients about the quantitative risks and benefits of any medical decision [12,13]. However, a study of actual primary care consultations found that qualitative rather than quantitative formats were used exclusively in 73% of consultations where CVD risk was discussed [14]. An international survey of physicians involved in primary prevention of CVD suggests that this may be due to the time required to calculate and explain quantitative absolute risk [7]. Broader risk communication research has also shown that percentage formats are poorly understood by both clinicians and patients, which may be a barrier to communicating quantitative CVD risk [15,16].

The aim of this study was to explore GPs’ descriptions of their communication strategies in CVD risk management, and investigate the reasons why they do or do not communicate quantitative absolute risk guidelines to patients.

## Methods

### Study design & setting

This was a qualitative study involving semi-structured interviews with 25 currently practising GPs from 8 Divisions of General Practice in metropolitan areas of New South Wales, Australia. The interviews were conducted between October 2011 and May 2012. Ethical approval was obtained through the Human Research Ethics Committee of the Sydney Local Health District.

### Recruitment

Purposive sampling was used to obtain a sample with a range of characteristics known to influence CVD risk management (see Table 1) [17-19]. Invitation letters were posted to members of Divisions of General Practice in metropolitan New South Wales. Fifty-five GPs returned expression of interest forms, of which 25 were allocated to this study, and 25 were allocated to another interview study focused on older adults, aiming for maximum variation in both studies. GPs signed a consent form before participating in person (n = 2) or via telephone (n = 23), and received $100 for their time. Preliminary analysis suggested saturation of key themes relating to the range of CVD risk communication strategies described by GPs, so no further recruitment was conducted [20].

**Table 1:** Characteristics of the 25 GPs interviewed

**Characteristic**	**Category**	**n**
**Gender**	Female	15
	Male	10
**Age (years)**	<40	6
	40-49	8
	50-59	7
	60+	4
**Years of practice**	<10	5
	10-19	6
	20-29	9
	30+	5
**GP role in practice**	Registrar/in training	1
	Contractor/sessional/retainer/salaried	14
	Partner/principal	10
**Medical record system**	Electronic only	23
	Electronic and paper	1
	Paper only	1
**Number of GPs in practice**	1-5	10
	6-10	13
	11-15	2

### Data collection

A semi-structured interview schedule covering CVD risk assessment and management was developed and piloted with 2 GPs. The final interview schedule included: 1) how they assess CVD risk generally, 2) whether they use absolute risk scores or not, and why; 3) how they communicate CVD risk to patients, 4) how they would manage specific cases, and 5) how they monitor and reassess patients. Audio-recordings were de-identified using participant ID numbers, and transcribed verbatim. Any references to identifying information in the transcripts were removed from the saved files. The interviews were conducted by two researchers trained in public health qualitative research methods (CB, SM).

### Analysis

A Framework Analysis method was used, which involves five steps and an iterative rather than linear process [21]. 1) Familiarisation with the data: CB, SM and JJ read through a subset of transcripts and independently identified themes using both deductive (researcher-driven) and inductive (response-based) methods. 2) Creating a thematic framework: CB, SM and JJ discussed the themes to develop a preliminary coding scheme, which was reviewed with KM to finalise the coding framework. 3) Indexing: CB and SM coded the remaining transcripts according to the framework, with new themes and revisions to the framework discussed. 4) Charting: CB and SM independently summarised themes and supporting quotes from each transcript in the framework (a matrix with participants as rows and themes as columns), with discussion to resolve any disagreement. 5) Mapping and interpretation: CB examined the framework within and across themes and participants to identify overarching themes and relationships, and the results were discussed with all authors. Rigour was addressed by: repeated coding of transcripts by different team members to ensure a comprehensive themes list and framework was achieved; an iterative process of constant comparison between the existing framework and new data; detailed documentation of the analysis process; and discussion of emerging and final themes with all authors, including two academic GPs (JD, PG) [22]. Results on GPs’ CVD risk assessment strategies that emerged from the same analysis process are reported separately [23].

## Results

### General communication

GPs’ description of their general communication style ranged from paternalistic to patient-centered. However, these styles were not mutually exclusive, as some GPs described both styles for different patient situations. For example, the following two quotes are from the same GP:

Paternalistic: *“I am a hard master, I’m a very scary person…and I won’t let you get away with things. But it’s only because I care and because I want good things for you.”*

Patient-centred: “*This is a partnership not a dictatorship so it has to be something that’s on your agenda as well as mine.” (ID24, female, 18 years experience)*

Communication was tailored to the GP’s perception of the patient’s : 1) level of CVD risk, which was perceived as low or high based on absolute risk assessment or more subjective methods [23]; 2) motivation to change lifestyle, ranging from resistant, to unmotivated, to determined; and 3) anxiety about CVD risk, ranging from dismissive, to concerned, to overly anxious. This resulted in three main communication strategies, summarised in Table 2.

**Table 2:** Communication strategies and perceived patient factors

	**Communication strategy**		
	**‘Positive’**	**‘Scare tactic’**	**‘Indirect’**
**GP perception of patient’s CVD risk**	Lower	Higher	Low but lifestyle risk factors
**GP perception of patient motivation**	Determined	Unmotivated	Resistant to change, do not understand risk
**GP perception of patient anxiety**	Concerned	Dismissive	Overly anxious
**Aim of communication**	Reassure and motivate patient	Scare patient into taking action	Avoid negative/confused reaction from patient
**How absolute risk is used**	Reassure that current risk is low; show achievable future risk reduction	Emphasise current high risk; show increased future risk of CVD event	Use alternative risk formats (e.g. qualitative description of risk level, colour-coded risk chart)

### Strategy 1: positive

A ‘positive’ strategy was used to reassure and motivate patients, with a focus on achievable changes they could make to reduce their CVD risk, using optimistic and encouraging language. This was considered particularly helpful for patients at lower risk of CVD, those who were concerned about their health but not overly anxious about it, and those who were willing to negotiate goals for lifestyle change and/or medication to address CVD risk factors.

“Reassuring people a bit and helping them to understand that they can control their risk factors either with or without medication and then I think that gives them a sense of empowerment, a bit of control.” (ID26, male, 20 years experience)

“I’m trying to convince them that they’re eating too much and not exercising enough and they’re trying to convince me that they are…but the ones that take it on board and make progress…they feel positive… encouraged… rewarded…motivated to keep going.” (ID36, male, 25 years experience)

In these situations, GPs used absolute risk to show patients how their CVD risk could be reduced by making changes to their lifestyle or potentially taking medication if lifestyle changes were not effective in reducing CVD risk factors. The focus was on their current risk if the patient needed reassurance that their risk was currently low, or on how their future risk would be lower if achievable changes were made.

“*The program I have got on my computer will show the impact of intervention… if you lower cholesterol for say 2 points and that would show them the improvement that they would get and lower their risk…they can see the improvement in that so it’s educational as well on the risk factors.” (ID37, female, 26 years experience)*

### Strategy 2: scare tactic

A ‘scare tactic’ strategy was used for patients who were considered to be at high risk of CVD, particularly males and smokers, those who were dismissive about their health, and those who were unmotivated to change their lifestyle.

“I like to…put a little fear into them…if they don’t ‘pull up your socks’ (sic) bad things can happen to them…if you don’t want that kind of scenario you do what I tell you.” (ID10, male, 40 years experience)

“Smoking is more dangerous for you than sticking yourself with an AIDS infected needle 3 times a year, 3 years in a row…so while you continue to smoke we will regard you as mad.” (ID36, male, 25 years experience)

In these situations, absolute risk charts and calculators were used by some GPs to ‘scare’ patients into taking action to reduce their risk of CVD, either through lifestyle change or medication. The focus was on the current level of risk already being high or elevated for their age, or on how their future risk would be even higher and potentially lead to CVD event.

“I can use it sometimes to sort of scare them a little bit. In the sense that, look your chances of having an incident in the next 10 years or 5 years is such so what do you want to do about it.” (ID5, female, 36 years experience)

### Strategy 3: indirect

An ‘indirect’ strategy was used when the GP was aware that CVD risk was an issue for the patient, but felt that a strong focus on discussing CVD risk in the consultation would be inappropriate or unhelpful. GPs described a number of reasons why they avoided explicitly assessing and/or communicating absolute risk in these situations.

GPs felt that patients who were very resistant to discussing CVD risk may not return if the issue was pressed, particularly male patients and smokers. In these cases, GPs brought up the issue of CVD but would stop the discussion if the patient had a negative reaction to the topic. Other patients had more important problems than CVD risk, either acute conditions that dominated one-off consultations or competing chronic issues such as mental health. In these situations, absolute risk was often not assessed until the patient was ready to discuss CVD risk.

“To be honest if I talk too much then they don’t turn up, they go to some other doctor. (laughter). It’s very true with male patients they don’t really want to find out what’s wrong with them unless they feel they need help.” (ID6, female, 19 years experience)

“Cardiovascular risk just isn’t on their agenda, they are more worried about their day to day social issues or their mental health issues even though technically in the back of my mind they’re more likely to die from a heart attack (than) from suicide or violence.” (ID16, male, 9 years experience)

In other cases, the absolute risk was assessed but not communicated directly to the patient because the GP found the percentage risk format to be unhelpful. This occurred when the GP perceived probabilities to be meaningless or confusing for the patient, for example those with limited education. However, other GPs described using alternative ways of explaining absolute risk in these situations, such as using colour-coded charts or graphs, or describing the risk level.

“A lot of the people that I see don’t have great levels of literacy and I think that’s one, that chart can be quite useful because you can look at the colours. But you need to just be really clear that you have explained how the chart works and how you’ve got to that because… if you’re not someone that’s used to looking at graphs, how the graph is set up can be a little difficult.” (ID14, female, 20 years experience)

GPs also avoided communicating absolute risk to low risk patients with lifestyle risk factors or high anxiety. If the GP wanted to motivate the patient to change their lifestyle, some felt that communicating a low percentage risk would undermine the behaviour change message. If the patient was highly anxious about their health, they may interpret even a low risk as something to be concerned about.

“Often people come out with a really low risk and then they think they (can) continue with their smoking…obesity…or borderline blood pressure. So if it comes out as 1 or 2% as a pretty low risk…I guess I am reassured but I was really trying to use it to warn them and maybe…change their behaviour.” (ID29, female, 25 years experience)

“Ones that have a high cholesterol just about freak out and they don’t need anybody more telling them…their risks of having a heart attack… I would be a bit dubious about showing them straight off because they would only get themselves into more of a state.” (ID7, female, 35 years experience)

### Risk formats

The formats used for risk communication varied widely depending on GP preference and patient factors, including both quantitative and qualitative descriptions of absolute risk. These are summarised in Table 3.

**Table 3:** GPs’ use of different CVD risk formats

	**Qualitative risk formats preferred**	**Quantitative risk formats preferred**
**Patient factors considered by GP**	- patients who don’t understand the numbers (‘blank look on face’)	- bring up numbers if patient is higher risk or risk not well controlled/ managed
	- numbers assumed to be less helpful for less educated/literate patients	- patients who are interested in the science/evidence (males, highly educated)
	- patients who will get stuck in a long discussion of the numbers rather than focusing on what they can do	- use numbers to justify treatment/no treatment for borderline patients
	- withhold absolute risk from highly anxious and low risk patients	- gamblers more familiar with probability
**Descriptions of risk**	- risk level: low/negative risk, medium/moderate risk, high/severe/positive/increased risk	- absolute risk % (probability of a heart attack or stroke in the next 5 or 10 years)
	- multiple risk factors: coexisting, mounting up, exponential	- convert absolute risk % into a frequency (e.g. 1 in 8 people like you)
	- scenarios: future cardiac event, being ill for a prolonged period, explain in terms of patient’s life (e.g. family member who had CVD event)	- change in absolute risk % if risk factors reduced
	- analogies: compare to other risks	- relative risk for particular populations (diabetics, high risk ethnicity)
		- compare individual risk factor results to guideline targets
**Communication tools**	- position on colour-coded absolute risk chart (red = high risk, green = low risk)	
	- absolute risk calculator to show current/future risk and effect of risk factors	
	- images (e.g. cholesterol spikes, what the brain looks like during a stroke, coronary artery to show relationship between high cholesterol and heart attack)	

Quantitative formats were conveyed in terms of the percentage risk of a CVD event and how this might change if risk factors were reduced, converting this to a frequency, explaining individual risk factor levels and targets, and sometimes the relative risk for the population to which the patient belonged. Such quantitative formats were described as most useful for high risk patients, those who demonstrated interest in the evidence and probabilities, and those who disagreed with the doctor’s recommendation. GPs identified males, highly educated patients, and gamblers as being more likely to show interest in and understanding of quantitative risk formats.

“Probably patients that…would like more of an idea what percentage risk they are of having a cardiac event in the next 5 years or whatever. Probably more....males generally I would imagine I’ve done it on.” (ID8, female, 23 years experience)

“I think people with a higher education level are much more interested in perhaps in absolute figures and like to see the chart or the risk calculator and see how things can change. Whereas if you’ve got…someone who is less educated then you need to be a little bit more…simplistic in your description of risk and changing risk.” (ID31, male, 30 years experience)

“You’ve got a 5% risk of having a heart attack or whatever. You know so if there were 100 people in this room 5 of them would have that event, so it’s just trying or you know if there were 20 people then there is 1 so that would be 1 in 20 chance. It depends, sometimes people kind of get it, particularly if they do a bit of gambling.” (ID14, female, 20 years experience)

However, some GPs felt that patients didn’t understand quantitative absolute risk formats.

“Basically you tell them that they’ve got a 10%, so 1 in 10 people…that have your profile will end up potentially having a heart attack or a stroke, so that’s the way I would explain it…but I think it is difficult for most people to sort of understand what that means.” (ID32, male, 6 years experience)

Qualitative formats were used if the GP felt the patient would understand this better than a quantitative format, based on their reaction to quantitative information or the assumption that less educated or literate patients wouldn’t understand. Qualitative formats were also used for highly anxious patients. The level of risk was described as low, medium/moderate or high. High risk was described in more varied ways using words like severe, positive and increased, or describing multiple risk factors as coexisting, mounting up, or exponential.

“I go through the individual risks and just say like it’s mounting up and it’s exponential…mild, moderate or severe.” (ID8, female, 23 years experience)

The consequences of high risk were described using scenarios, such as a period of prolonged illness or death. Some GPs used visual tools to emphasise the potential severity of CVD risk, such as pictures of atherosclerosis and damage caused by CVD events. Another approach was to use analogies comparing CVD risk to other risks that they knew the patient would not be inclined to take.

“I show them what it means to have a stroke because I have pictures of the brain with haemorrhages.” (ID5, female, 36 years experience)

“I give them an example like if you jump from a roof and you expect you are not going to get hurt, are you silly or not? You will get hurt, to what extent you will get hurt really depends on a lot of factors. Whether you end up having a skull fracture or multiple fracture or just a small fracture that depends but you will definitely get hurt.” (ID38, male, 45 years experience)

## Discussion

The results of this study provide an insight into how GPs use absolute risk within broader communication strategies about CVD risk, and how this may influence the use of absolute risk assessment. Using a ‘positive’ or ‘scare tactic’ strategy could facilitate the use of absolute risk assessment for communication purposes, while an ‘indirect’ strategy discouraged both the assessment and communication of absolute risk. However, some GPs found it helpful to use non-quantitative absolute risk formats within an ‘indirect’ strategy. A challenge for the implementation of absolute risk guidelines is finding a way to make absolute risk assessment useful regardless of the communication strategy being used.

GPs in this study adapted their communication approach based on their perception of the patient’s risk, motivation and anxiety. Absolute risk could reinforce a ‘positive’ strategy for lower risk and motivated patients, or a ‘scare tactic’ strategy for higher risk and unmotivated patients. However, it was seen as inappropriate for less educated patients, and lower risk patients with high anxiety or low motivation, resulting in an ‘indirect’ strategy. As identified in previous research, the concept of absolute risk was sometimes considered difficult to explain [8,9]. However, some GPs demonstrated how alternative absolute risk formats could be used in these situations, such as the use of colour-coded charts and graphs, and guideline-based qualitative descriptions of the risk level as low, moderate or high. Best practice guidelines for risk communication suggest that alternative risk formats may be beneficial for patient understanding, including verbal and visual formats, but their benefit may depend on patient characteristics [13,24]. In line with this, a recent qualitative paper on alternative absolute CVD risk formats found that patients preference for and understanding of equivalent verbal, numerical and visual formats was very variable [25]. Our finding that male patients were perceived by GPs to be more interested in the evidence, prompting communication of quantitative absolute risk, provides an explanation for a previous study’s finding that qualitative formats were used more for female patients [14], but further quantitative research is needed to identify the best risk formats for different patient groups [13].

Although shared decision making is increasingly accepted as an ideal approach [12], discussions of CVD risk that do not provide information in ‘optimal’ quantitative risk formats may instead be focusing on one of the other functions of medical communication. These include fostering the relationship, gathering information, providing information, decision making, enabling disease and treatment related behaviour, and responding to emotions [26]. A ‘positive’ strategy might be more focused on fostering the relationship than providing information, a ‘scare tactic’ strategy may be required to enable disease and treatment related behaviour, and an ‘indirect’ strategy may involve gathering information about competing concerns and responding to negative emotions regarding CVD risk.

More broadly, this study sheds light on how GPs reconcile the sometimes conflicting aims of evidence-based guidelines and patient-centred care, by adapting their communication approach based on psychosocial factors. Our findings suggest that GPs’ perceptions of patients’ risk, motivation and anxiety determine whether GPs use a shared decision making approach, rather than a general tendency to use shared decision making versus paternalistic styles across all patients. This is in line with another qualitative study that found GPs ‘decide who decides’ depending on the level of risk and anxiety of the patient [27]. If the patient was judged to be lower risk, the GP was more likely to actively involve them in treatment decision making, unless they were perceived as being too anxious to decide. However, GP and patient perceptions of CVD-related risk and anxiety may differ considerably [14], and GP judgments of overall CVD risk are not necessarily consistent with calculated absolute risk [28]. The shared decision making approach also requires a distinct point at which the need for a decision is identified, after which quantified risks and benefits can be considered before choosing one of several options [12,13]. This may not always be applicable to the management of CVD risk, since the benefits and harms of lifestyle versus medication approaches for a particular patient change over time, as individual risk factors increase or decrease.

Future research could investigate education approaches that equip GPs with a range of ways they can describe and explain absolute risk, including quantitative, qualitative and visual approaches that are consistent with the guidelines. The New Zealand Heart Foundation has implemented such a tool, which includes percentage risk, an equivalent ‘heart age’ format, a graph showing how risk will increase with age, and colour-coded risk levels, but its efficacy has not been formally assessed [29,30]. Taping real consultations to more objectively analyse the communication strategies identified in this study, as well as the impact of alternative absolute risk formats, would also be beneficial.

The strengths of this study include a heterogeneous sample and rigorous analysis process. The findings are limited by the reliance on self report, as GPs’ descriptions of their communication approaches may not match what they actually do in practice. Taping consultations could address this issue. Our method did however allow GPs to reflect on a wide range of patient situations they had encountered, and explain why their communication strategies and use of absolute risk may differ across patients.

## Conclusions

GPs in this study described tailoring their communication approach based on their perception of each patient’s risk, motivation and anxiety, resulting in three distinct CVD risk communication strategies: ‘positive’, ‘scare tactic’, and ‘indirect’. The findings demonstrate how alternative formats for absolute risk can be useful within each of these communication strategies. Providing GPs with different ways to explain absolute risk, in order to achieve different communication aims, may improve their use of absolute CVD risk assessment in practice.

## Abbreviations

CVD: Cardiovascular disease; GP: General Practitioner.

## Competing interests

The authors declare that they have no competing interests.

## Authors’ contributions

CB contributed to study design, recruitment, data collection, framework analysis, interpretation, drafting and revising the manuscript. JJ contributed to study design, framework analysis, interpretation, and revising the manuscript. SM contributed to recruitment, data collection, framework analysis, interpretation, and revising the manuscript. LI contributed to study design, interpretation, and revising the manuscript. JD contributed to study design, interpretation, and revising the manuscript. PG contributed to study design, interpretation, and revising the manuscript. KM contributed to study design, analysis and interpretation, and revising the manuscript. All authors read and approved the final manuscript.

## Authors’ information

Authors’ qualifications and position titles: CB (MPH; PhD Candidate), JJ (PhD; Research Fellow), SM (MIPH; Research Assistant), LI (PhD; Professor), JD (MBBS, PhD; Professor), PG (MBBS, PhD; Professor), KM (PhD; Associate Professor).

## Pre-publication history

The pre-publication history for this paper can be accessed here:

http://www.biomedcentral.com/1471-2296/15/106/prepub
